# Periscope: Pathology and Therapeutics

**Published:** 1818-07-01

**Authors:** 


### FOURTH DEPARTMENT.

THE

### Quarterly Periscope,

OR

SYNTHETICAL VIEW OF THE BRITISH JOURNALS,

WITH

*PRACTICAL COMMENTARIES.*

[ JANUARY, FEBRUARY, MARCH, 1818. ]

Nihil est aliud magnum quam multa minuta.

### I.

### *Pathology and Therapeutics.*

1. *Continued Fever.* Mr. Edmonston has written a long account of a fever lately prevalent in Newcastle-upon-Tyne [*Ed. Journ.* 53.], which appears to have been typhus mitior, and unaccompanied by any remarkable peculiarity. "In all," says he, "the failure of strength, and the loss of flesh, were very conspicuous, even when the complaint was mild and of short duration." The general method of treatment was *depletory,* although the varying features of the disease required, of course, a corresponding variety of cure. The duration of the epidemic did not exceed six weeks, and it left scarcely any sequelæ behind. Its character of contagion was not exactly ascertained by the author.

A more energetic practice is detailed in the January Number of the *Medico-Chirurgical Journal* in *typhus,* as it occurred in Scotland. Blood-letting, purgatives, cold or tepid lavations, with calomel, conducted to a favourable termination, in almost every case. "Calomel, says the writer, was exhibited, not only to preserve a regular state of the bowels, but by its specific action, to modify or change the diseased secretions and excretions. That it did so, was evidently proved by inspection of the alvine evacuations, which at first, were commonly thin, fœtid, and ill conditioned; but gradually became of a light yellow colour, and changed from that to a dark brown on the specific effects of mercury becoming visible. The calomel was generally given at the commencement of the attack, in the proportion of a *scruple for a dose,* with the view of evacuating the bowels, which it seldom failed to effect in the course of eight or twelve hours after its exhibition, producing a few copious dejections, without much fatigue or irritation. Subsequently, it was administered as an *alterative,* in small doses, combined with opium." It is somewhat curious, that the declaimers against mercury, are generally those whose prepossessions prevent them from giving it a trial in typhoid fevers. It was precisely the same with blood-letting. Dreadful were the effects of this remedy in the hands of those —— who never employed it; while no complaints were made by those who were every day bleeding, *pleno rivo!*

Dr. Moulson, Physician to the Halifax General Dispensary, has, in the February Number of the same Journal, made some interesting remarks on typhus. Whenever *head-ache* was violent, the prostration of strength was disregarded, and the temporal artery was opened. If that could not be done, eight or ten leeches were applied. From experiments, Dr. M. is led to believe, that arteriotomy is much less debilitating than venesection. If the bowels were confined, he opened them by calomel and jalap; if otherwise, he proceeded on the following plan: three grains of calomel, one of antimonial powder, and half a grain of opium, were taken in a pill, every four hours, with two table spoonsfull of a mixture composed of infusion of roses, sulphate of magnesia, and carbonate of magnesia. So soon as the mouth became affected, generally on the third or fourth day, the bad symptoms gradually vanished.

*Typhus, attended with Trismus and Amaurosis.*—Mr. Atbury has recorded [*Medical Repository, No.* 49], a case of trismus occurring in an irritable female, affected with a typhoid fever, brought on by cold and suppressed catamenia. The trismus came on the eleventh day of the fever, and lasted three days. Ptyalism did not remove the spasm, but electricity was more successful. Amaurosis supervened six days afterwards, from which she slowly recovered.

Mr. Webster, Surgeon of the 51st Regiment, has [*Medico Chirurgical Journal,* 27.] made some valuable remarks on the influence of climate and atmospheric vicissitudes in varying the features of fever. He has added his mite to the crowd of facts which have, of late years, attested the superiority of depletion in typhus and other fevers.

2. *Fever, Remittent.* A communication on the subject of Remittent Fever, as it appeared in Cephalonia, is given by Dr. H. Robertson, in the 50th Number of the *Repository.* It broke out in the summer of 1816, among the Garrison of Argostoli, and resembled the Causus of the West, destroying a third of those attacked. The local affections were, in general, confined to the abdominal viscera, the head being rarely engaged. Constipation was uncommon; delirium occurred in only one case. It affected in preference, those lately from England, and of plethoric constitutions. As the fever exhibited " tertian accessions" the bark was tried, but without success, as the gastric irritability was extreme. Owing to crowding, &c. the air became so vitiated, that not only every case, immediately after admission, became aggravated, *but all persons having communication with the hospital were taken ill.*"

A more interesting paper on this subject is from the pen of Dr. Boyd [*Medico-Chirurgical Journal,* 25.] in a Report of Occurrences on board the Hospital-ship Gorgon on the expedition to New Orleans in 1815. While the troops touched at Barbadoes, the ardent fever visited those exposed to atmospheric influence, intemperance, &c. and showed the usual character of tropical fever. It was ably combated by copious abstractions of blood, purgatives, abstinence, " with the exhibition of calomel, in the quantity of six, eight, or ten grains every four hours, either by itself, or combined with small doses of opium and antimonial powder, according to circumstances, with an occasional purgative. This mode of treatment had the happiest effect in conducting the disease to a favourable issue." Dr. B. is certain, that blood drawn from the temporal artery afforded more relief to the head than venesection; but he is not sure the effects are more permanent. Dr. Forbes, of Penzance, [*Med.-Chirurg. Journal,* 26] has also thrown out some valuable practical hints on the etiology and treatment of Tropical or Remittent Fever, in a report of health from on board H.M.S. Venerable, in the West Indies. The depletory practice was boldly but judiciously employed, and with the happiest effect, when used early. In a smart epidemic of fever and dysentery, which brushed the crew of the Venerable, in Freeman's Bay, Dr. F. traced the causes to "severe labour in the heat of the sun, and checked perspiration from sudden gusts of wind." He satisfactorily proves that these, and *not miasmata*, gave origin to the fever and dysentery. We entirely coincide with Dr. Forbes, and are quite satisfied that many epidemics have owed their origin to these causes, when miasmata were blamed for all. Dr. F's *Meth. Medend.* was strictly depletory; Dr. Crichton's, at the hospital, inclined to the depletory and mercurial, so as to affect the mouth, by calomel.

3. *Diseases of the Spinal Cord.* Dr. Sanders, of Edinburgh, has long and successfully investigated the pathology of nervous and spasmodic diseases; and from an excellent paper in the January Number of the *Medico-Chir. Journal and Review*, it appears that the grand seat of lesion is in the spinal marrow or medulla oblongata. Thus the appearances on dissection of an hydrophobic patient, as detailed by Surgeon Webster, of the 51st Regt. are, says Dr. Sanders, "precisely of the same kind with those which I have uniformly found in *trismus, asthma, pertussis, croup*, and all *spasmodic affections*, where the more remarkable effects are produced from the mouth to the diaphragm inclusively." Dr. S. observes, that in all deaths from croup, whether of the spasmodic or inflammatory species, effusion more or less is always found in the cerebellic cavity, and at the top of the spine, as well as turgescence of the veins surrounding the medulla oblongata and the nerves thence arising. This range of morbid action internally is denoted, during life, by a corresponding extension of morbid signs: so intimately, in effect, are these connected—so exactly do the symptoms indicate the locality of the distending vessels on the nervous system, that before the opening a body, Dr. S. always prognosticates the appearances unfolded by the scalpel, and is rarely mistaken. Thus a child died of a very short illness, and the head, thorax, and abdomen being examined, it was reported that no cause of death could be found. But the spinal canal being opened, by sawing through the *bodies* of the vertebræ from within, the spinal marrow, its nerves and envelopes, were found diseased from the atlas to the sacrum. The dura matral covering was of a dark crimson colour, and surrounded with coagulated lymph; serum filled the canal; the sinus venosi were dark and distended.

It is well known that Dr. Sanders has taught these important doctrines for very many years in Edinburgh; and yet on the very same day that the above paper appeared, a laborious disquisition on disorders of the spinal cord appeared in the *Edinburgh Journal*, No. 53, by Dr. Abercrombie, in which disquisition the name of Sanders is never mentioned! Without attempting to unfold the reason of this mysterious transaction, we shall proceed to the disquisition itself.

Dr. A. relates a case of *inflammation* and *suppuration* of the spinal cord, and quotes several others from various authors, the symptoms of which were sufficiently indicative of the evil. After treating on serous effusion, induration of the cord, thickening of the membranes, &c. our author comes to increased vascularity and turgidity of the vessels of the spinal cord and its membranes. This *plethora spinalis* has been considered by continental physicians as the cause of disturbance in various functions of the human frame, as tremors, convulsions, paralytic affections, chorea, epilepsy, and tetanus, in consequence of the irritation at the origin of the spinal nerves. And it is *now* beginning to be proved, that the *conjectures* of these physicians were founded in truth, as the foregoing quotations from Dr. Sanders will evince, notwithstanding the following scepticism of Dr. Abercrombie:—" It is, however, extremely doubtful, whether *turgidity* of vessels can with propriety be considered as a cause of disease." 56. We conceive that *turgidity of vessels* in such structures as the brain and spinal marrow, must be a very fruitful source of disease; and that the said turgidity is, in fact, the immediate precursor of still more dangerous states—inflammation and effusion. This is proved by the very cases which Dr. A. brings forward himself, of which we may notice the *third.* " A young man had fever and high delirium. When the delirium subsided, he had convulsive motions of the *superior* extremities, and soon after died comatose. On *dissection*, the vessels of the pia mater of the spinal marrow, at its *upper* and posterior part, were found *distended* with blood, as if they had been highly injected. This was especially remarkable about the origin of some of the spinal nerves. There was a similar appearance on the pia mater of the *brain*, and some serous effusion on its surface." *Morgagni.*—Surely no case can more firmly establish the mutual connexion between the nervous and vascular systems than this; or more plainly shew the decisive influence which they reciprocally exert on each other. To what were the coma and convulsive motions of the superior extremities owing, but to turgescence of the vessels on the brain and spinal marrow?

Dr. Abercombie next comes to a division of his paper, entitled "CONJECTURES," which, however, he thinks, are not to be altogether overlooked. Hoffman attributed *epilepsy* to affections of the membranes of the brain, and *convulsions* to those of the spinal marrow. Ludwig attributes hypochondriacal and hysterical affections to irritation of the origins of the intercostal nerves, and Lieutaud holds a similar doctrine.

M. Esquirols asserts, that he opened the bodies of fifteen people who died of *epilepsy*, and found the spinal cord affected in all the cases.

That *tetanus* was caused by affections of the spinal marrow, we have the authority of Burserius, who relates the case of a tetanic patient, in whom a large quantity of yellow serum was found under the outer covering of the spinal marrow. A decisive case of *tetanus*, from affection of the spine, has escaped the researches of Dr. Abercrombie. M. Dupuytren lately opened a man who died of *traumatic* tetanus, exquisitely marked, where the coverings of the spinal marrow were intensely red, which redness extended to the coverings of the nerves, as they emerged from the theca vertebralis.—*Journ. de Med.*

M. Salin *conjectured* that *hydrophobia* might be owing to affections of the spinal marrow; and the late remarkable case, recorded by Mr. Webster, has gone far to prove his conjecture well founded. Upon the whole, we have ample reason for prosecuting this interesting investigation, and hope that Dr. Sanders and his pupils will continue with that unabated ardour which conquers difficulties by daring to oppose them.

4. *Aneurism of the Heart.* Dr. Carbutt, of Manchester, has recorded [*Annals of Medicine,* No. 8.] another of these melancholy cases which now are becoming so frequent. A woman, aged 42, was admitted into the Manchester Infirmary with the following symptoms:—Heart beats over an extended surface; pulse rapid, weak, irregular, and hardly perceptible at the wrist; undulating pulsation in the neck, on the right side, in the course of the internal jugular. Respirations 30 in the minute, and laborious; has spat blood; countenance puffy, pale; lips blue; inability to lie down; left arm benumbed; feet and legs œdematous; urine scanty, turbid, and of an orange colour; bowels irregular; wakes from sleep with oppression and anxiety. Has long felt great difficulty in walking up an ascent, or against the wind. The disease has gradually approached, but has increased rapidly within the last two months. She died in about a fortnight.

*Dissection.*—Surface of the body yellow; forty-eight ounces of bloody serum in the right thoracic cavity; in the left eight ounces; in the pericardium six ounces. Liver diseased and granulated; spleen indurated. Heart twice its natural size, with patches of coagulable lymph on its surface. Inferior cava greatly enlarged. The tricuspid valve was remarkably tied down, so as to perform its function imperfectly. The similunar valves diseased; coronary arteries enlarged. This is one of those unhappy combinations of Hepatic and Cardiac disease; the former greatly aggravating the latter. We have seen all the above-mentioned symptoms, however, most exquisitely simulated by functional or nervous irritation, and entirely disappear, after every one had pronounced the case desperate and incurable.

5. *Spasmodic Affections.*—Mr. Webster, Surgeon of the 51st Regt. has [*Med. Chir. Journ.* 27.] stated some curious and interesting cases of spasmodic diseases removed by active depletion. Case—A man was attacked with violent spasmodic pains in the abdomen, recurring at intervals, and accompanied by vomiting. Venesection, *ad syncope,* instantly removed the pain, and it did not return. Other cases of a similar nature are related. "Henceforth, says Mr Webster, I shall look upon the lancet as a most powerful *antispasmodic.*"

